# Investigating the genetic determination of duration-of-fertility trait in breeding hens

**DOI:** 10.1038/s41598-024-65675-0

**Published:** 2024-06-27

**Authors:** Wei Luo, Xishi Huang, Jingxuan Li, Lantao Gu

**Affiliations:** Institute of Biotechnology of Guilin Medical University, Guilin, Guangxi China

**Keywords:** Breeding hens, Duration-of-fertility, Fertile egg production, GWAS, Molecular markers, Genetics, Agricultural genetics, Animal breeding

## Abstract

The duration-of-fertility (DF), which was defined as the number of days when breeding hens lay fertile eggs following copulation or artificial insemination (AI), is an important economic trait in chick production when it has strong effects on fertile egg output and production costs. Little is known about the underlying genes and molecular markers related to DF trait to date. Here, we measured the DF of 701 Chinese Jinghong hens and 408 Jingfen hens. The DF showed high individual variability and potential for genetic improvement. Then, 192 Jinghong breeding hens were provided for a genome-wide association study, 27 SNPs respectively located in three genomic linkage regions (GGA1:41Kb; GGA3:39Kb and GGA8:39Kb) were suggested to be significantly associated with DF. Particularly, 6 of these 27 SNPs were further verified to be associated with DF in the 701 Jinghong and 408 Jingfen hens using PCR-RFLP genotyping method. These 27 SNPs were also mapped to 7 genes according to their genomic position. Furtherly, 5 of these 7 genes were tested using qPCR. Results show that the *CYP2D6, WBP2NL, ESR1* and *TGFBR3* mRNA expression levels of hens with long DF were significantly higher than the hens with short DF (P < 0.05). Overall, findings in our research provide new insight into the genetic basis of duration-of-fertility in breeding hens while providing new clues for further functional validation on the DF-related genetic regulation mechanism and improvement of DF through chicken breeding.

## Introduction

Following copulation or artificial insemination (AI), female fowls have the ability to store sperm in their reproductive tracts and then lay fertile eggs for days or weeks (depending on the species)^1^. This persistent period of laying fertile eggs was identified as the “duration-of-fertility” ^2^. It is a determinant limitation by which the frequency of AI must be managed in hatching egg production. Improvement in the duration-of-fertility could increase the interval between successive AI and thus reduce the number of raised breeder males and labor costs associated with AI^3^. Meanwhile, it could alleviate the stress and suffering of hens from frequent AI^4–6^. Therefore, studying the molecular mechanisms regulating the duration-of-fertility trait can help develop a better understanding of this trait and improve the economic efficiency of and animal welfare for the production of hatching eggs. When researching the duration-of-fertility, a measurable phenotype value, the number of days following insemination until the last fertile egg was produced, was most commonly used for reflecting this trait in hens^4,7, 8^. It is believed that the problems associated with the duration of fertility are both genetic and environmental factors, including diet, lighting conditions, age, feeding, and insemination methods, which could modulate the duration-of-fertility in hens^9–15^. In ducks, duration-of-fertility trait-related heritability between 0.15 and 0.24 has been reported by Brun et al.^16^, who analyzed fertility traits following crossbreeding by AI with pooled semen. Moreover, traditional selection trials have been used to produce generations with a long duration of fertility, and the possibility of increasing the AI intervals by improving the duration-of-fertility trait has been demonstrated. Although previous experiments showed that genetic improvement of DF is possible, it has not received much attention in genetic studies and commercial breeding programs compared with the intensive selection for egg and meat production traits. The development of genomic selection provides a rapid and effective method for the long DF hens breeding. However, little is known about the molecular markers and genetic mechanisms associated with duration-of-fertility trait to date. Genome-wide association studies (GWAS) take advantage of a large number of SNP markers in population-wide linkage disequilibrium with extremely narrow regions potentially harboring candidate loci for the complex traits^17–19^.

In the present study, with the use of a 600K Affymetrix Axiom High Density (HD) chicken genotyping array^20^, we performed a GWAS to uncover the critical SNPs or genes that affect the duration-of-fertility trait in hens using egg production data from Jing Hong chickens. This study is the first to conduct a GWAS of duration-of-fertility trait at the hen laying period.

## Results

### Description of the duration-of-fertility trait in breeding hens

**Figure 1:**
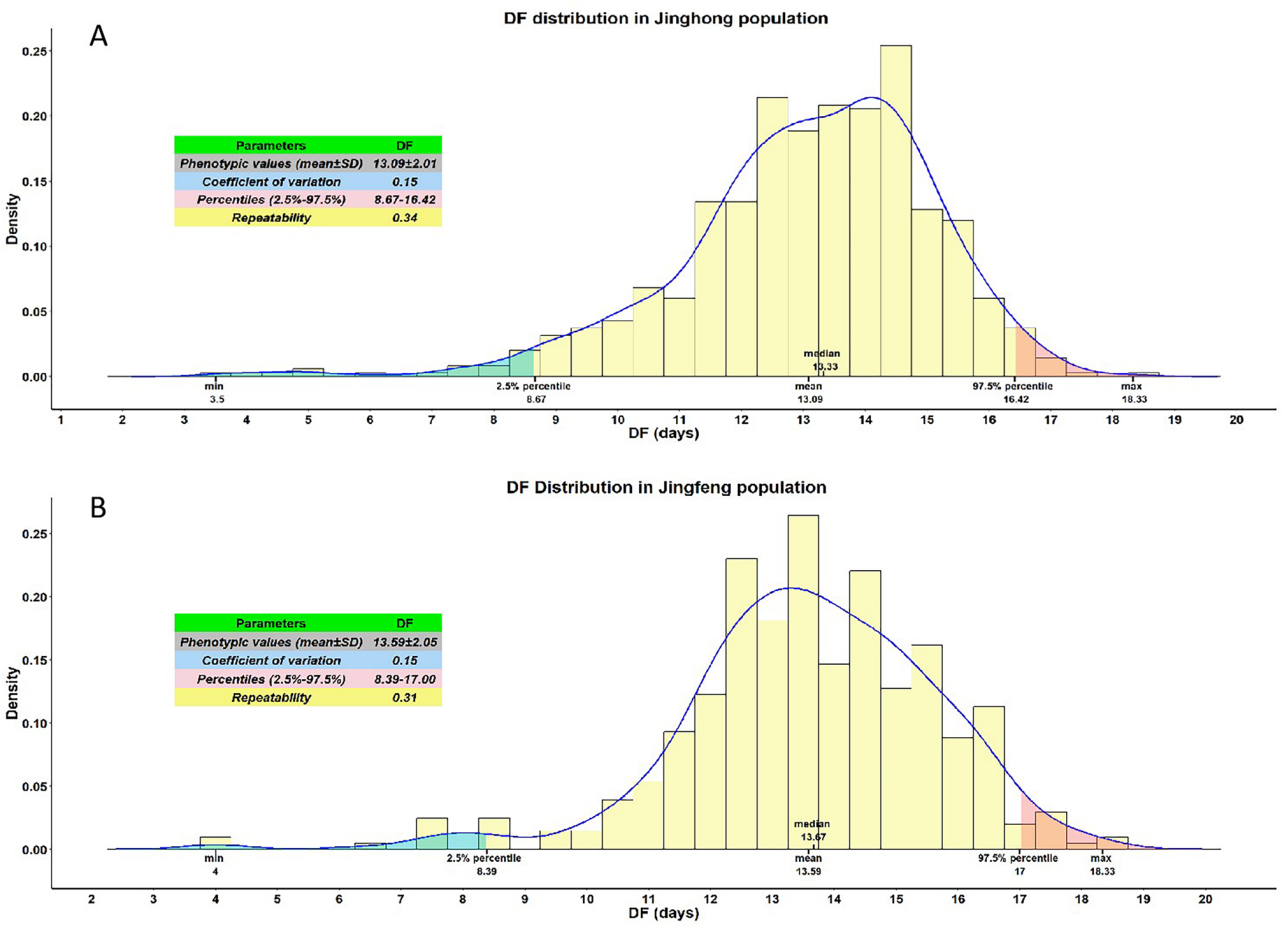
Description of the duration-of-fertility trait (DF, the number of days following insemination until the last fertile egg was produced) in (**A**) Jinghong population and (**B**) Jingfen population.

The duration-of-fertility trait (DF, the number of days following insemination until the last fertile egg was produced) was measured and analyzed in the Jinghong population and Jingfen population respectively. Their characteristics—including the phenotypic values (mean ± standard deviation), coefficient of variation, repeatability, and percentiles—are presented in Fig. 1. The DF shows high individual variability and follows normal distribution both in the Jinghong population and the Jingfen population (Kolmogorov–Smirnov test P > 0.05).

### 27 SNPs in chromosome 1, 3 and 8 were associated with duration-of-fertility by GWAS

**Figure 2:**
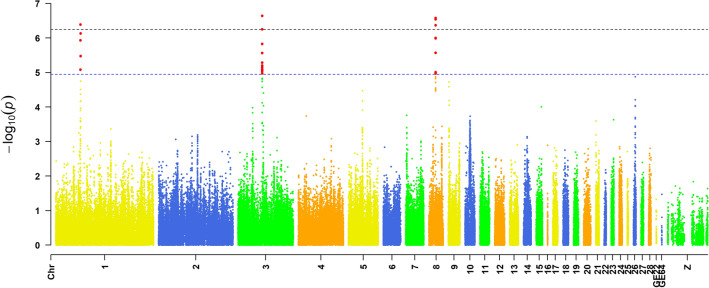
Manhattan plot of genome-wide association study on duration-of-fertility trait DF in breeding hens. The associated values of − log_10_(*P value*) are shown by chromosomes. The black highlighted line indicates the threshold of 5% Bonferroni genome-wide significance (*P* < 2.09 × 10^−7^), and the blue line indicates the significance level of “suggestive association” (*P* < 1.12 × 10^−5^).

Association tests for DF were performed using a univariate linear mixed model. There was no dramatic deviation between observed and expected − log10 (*P* value) in the Q–Q (Quantile–Quantile) plot (Fig. S1), suggesting that there was little or no evidence of residual population structure effects in test statistic inflation. The global view of *P*-values for all SNP markers was visualized by a Manhattan plot, as shown in Fig. 2. Here, a total of 6 SNPs reached the genome-wide significant level (*P* value < 5.62e−07) and 21 SNPs reached the significance level of “suggestive association” (P value < 1.12e−05) when performing GWAS. Subsequently, the proportion of phenotypic variance explained (PVE) by the combined effect of 27 suggested SNPs was assessed to be 0.662. These SNPs on GGA1, GGA3 or GGA8 could serve as new candidate genetic markers for the DF trait, and their genomic locus could serve as candidate QTLs for the DF trait.

### Annotation and polymorphism evaluation of candidate SNPs

The genomic locus and information of SNPs were obtained based on the ICGSC annotation of the Gallus gallus genome version 7.0 (NCBI RefSeq assembly: GCF_016700215.2). Among these 27 candidate SNPs implied by GWAS (Table 1), five SNPs were located within a 55.94 Kb genomic region that spans from 49,448,847 to 49,504,783 bp on GGA1; thirteen SNPs were located within a 156.68 Kb genomic region that spans from 48,604,632 to 48,761,316 bp on GGA3; nine SNPs were located within a 30.77 Kb genomic region that spans from 14,080,338 to 14,111,112 bp on GGA8. The *CYP2D6, WBP2NL, SEPTIN3, CENPM, CCDC170, ESR1* and *TGFBR3* genes are located within, upstream or downstream of these above three genomic regions. They could serve as new candidate genes for the DF trait, yet their roles need to be verified in further studies.

**Table 1:** Annotation of genome-wide significant SNPs associated with duration-of-fertility trait.

rs#	Chr.	Pos.	ALT/REF	MAF	PVE	P value	Consequence	Candidate/nearest gene
rs317323141	1	49448847	G:A	0.41	0.17	4.09E−07	intron1	CYP2D6 (NC_052532.1)
rs312949642	1	49454509	C:T	0.47	0.15	1.17E−06	Exon8	
rs14822125	1	49476575	A:C	0.47	0.13	8.23E−06	intron3	WBP2NL (NC_052532.1)
rs315957233	1	49491109	G:A	0.39	0.16	7.36E−07	intron1	SEPTIN3 (NC_052532.1)
rs13864590	1	49504783	A:G	0.40	0.14	3.34E−06	intron5	CENPM (NC_052532.1)
rs312853002	3	48604632	C:T	0.43	0.13	6.28E−06	Intron7	CCDC170 (NC_052534.1)
rs317270113	3	48640679	A:G	0.36	0.13	8.09E−06	intron1	ESR1 (NC_052534.1)
rs14356921	3	48721974	G:A	0.36	0.16	5.56E−07		
rs314642908	3	48722914	G:T	0.42	0.16	2.72E−06		
rs314819424	3	48730125	T:C	0.35	0.15	1.49E−06		
rs16273429	3	48733567	G:A	0.35	0.14	5.17E−06		
rs15353101	3	48736974	G:A	0.35	0.13	7.02E−06		
rs314157932	3	48741357	T:G	0.36	0.17	2.27E−07		
rs315718731	3	48741561	C:G	0.35	0.13	7.09E−06		
rs14356931	3	48748022	C:T	0.34	0.13	9.11E−06	intron2	
rs316568105	3	48757616	C:T	0.35	0.15	8.17E−06		
rs316246997	3	48760724	T:G	0.34	0.13	8.22E−06		
rs316694205	3	48761316	G:T	0.34	0.13	1.05E−05		
rs314887106	8	14073077	C:T	0.27	0.14	2.69E−06	upstream_5.41Kb	TGFBR3 (NC_052539.1)
rs315680042	8	14073653	C:T	0.29	0.17	2.65E−07	upstream_4.84Kb	
rs15913691	8	14076793	C:A	0.28	0.17	4.29E−07	upstream_1.70Kb	
rs313092228	8	14080338	C:T	0.29	0.17	2.88E−07	intron1	
rs14643191	8	14085227	G:A	0.28	0.16	1.03E−06		
rs317416878	8	14097079	G:A	0.28	0.13	9.90E−06	intron2	
rs314144090	8	14103506	A:G	0.27	0.16	9.99E−07		
rs316044128	8	14107436	T:C	0.28	0.13	9.78E−06		
rs316687055	8	14111112	C:T	0.29	0.13	1.07E−05		

ALT/REF, alternative allele/reference allele; MAF, Minor allele frequency; PVE, phenotypic variance explained.

**Figure 3:**
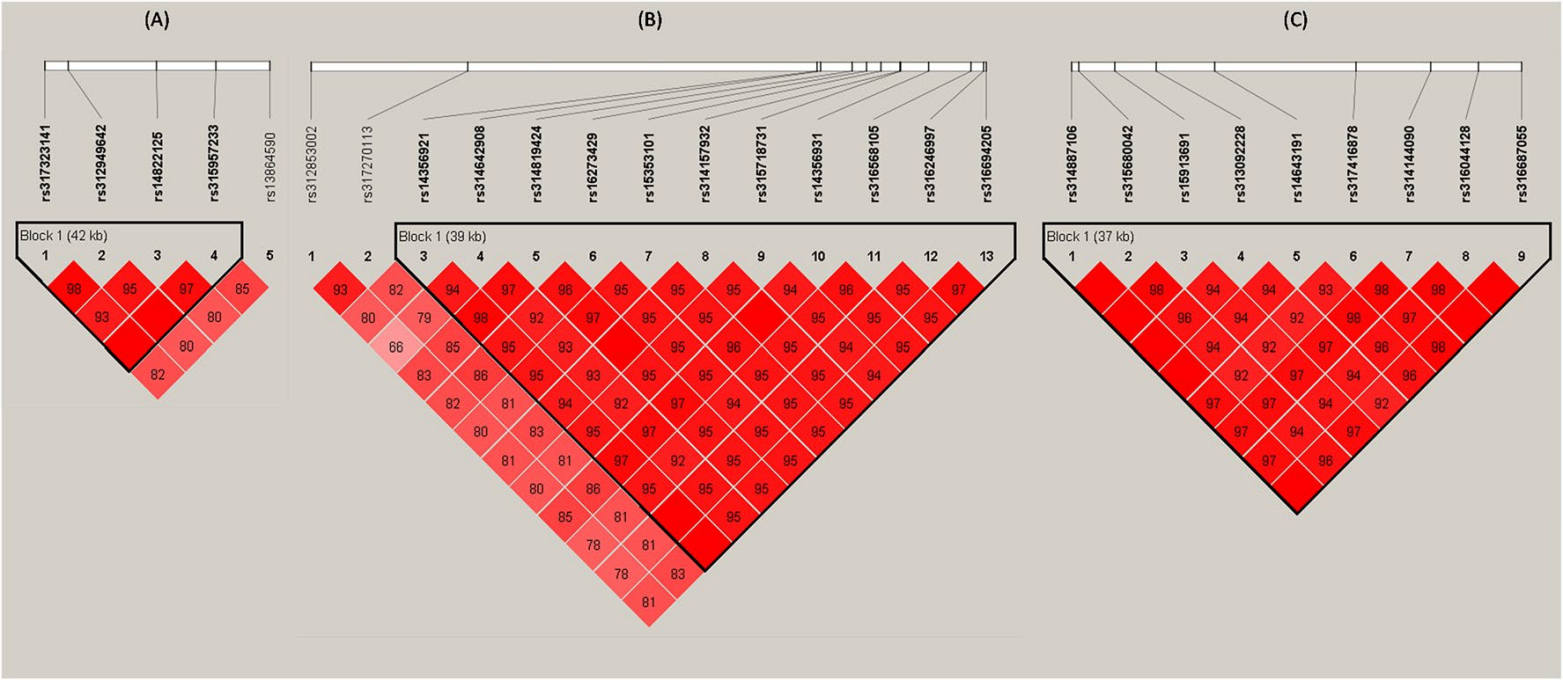
(**A**–**C**) Linkage disequilibrium (LD) of single nucleotide polymorphisms (SNPs) in chromosome 1, 3 and 8. Pairwise correlation (D′) values are shown between polymorphisms. The block’s color indicates the LD status of SNPs; deep red means high linkages between 2 SNPs. The haplotype blocks were defined by using the default setting of the Haploview software.

Haplotype blocks and LD structures were generated from the 27 significant SNPs genotyped in chromosome GGA1, GGA3 and GGA8 from the chicken population. LD analysis revealed that all genome-wide significant SNPs located in the above three chromosome regions were in strong LD status. 4 of 5 SNPs on GGA1 were clustered into a 42 Kb block, 11 of 13 SNPs on GGA3 were clustered into a 39 Kb block, 9 SNPs on GGA8 were clustered into a 39 Kb block (Fig. 3A–C).

### Six tag SNPs were further identified to be associated with duration-of-fertility

The genotype patterns of the 6 selected significantly associated SNPs by GWAS were further genotyped for validation using a PCR-RFLP approach, and association studies were performed with DF in the 701 hens of JHP and the 408 hens of JFP to test the significance of the difference of genotype effect on DF.

The genotypic and allele frequencies were calculated by observing the presence of various RFLP patterns. Association analysis results were shown between the SNP genotypes and DF in the chickens, and the means and standard error (mean ± SD) of different genotypes for each SNP are listed in Table 2. This follow-up validation experiment revealed that these 6 SNPs were all significantly associated with DF (P < 0.01). It was demonstrated that the association of GWAS SNPs with duration-of-fertility trait was reliable.

**Table 2:** Association analysis of 6 GWAS SNPs with duration of fertility trait DF in hens.

SNPs	Population	Genotype frequency (mean ± SD)			P-value
rs312949642		CC	CT	TT	
	P1	13.72 ± 1.97^a^ (165)	13.14 ± 1.84^b^ (342)	12.47 ± 2.17^c^ (194)	< 0.01**
	P2	14.21 ± 1.69^a^ (122)	13.85 ± 1.95^a^ (180)	12.43 ± 2.14^b^ (106)	< 0.01**
rs13864590		AA	AG	GG	
	P1	13.59 ± 1.89^a^ (234)	13 ± 1.96^b^ (309)	12.54 ± 2.13^c^ (158)	< 0.01**
	P2	14.09 ± 1.78^a^ (166)	13.73 ± 2.05^a^ (162)	12.26 ± 2.03^b^ (80)	< 0.01**
rs312853002		CC	CT	TT	
	P1	13.53 ± 1.82^a^ (200)	13.03 ± 1.96^b^ (330)	12.69 ± 2.23^b^ (171)	< 0.01**
	P2	14.34 ± 1.64^a^ (139)	13.44 ± 2.07^b^ (185)	12.68 ± 2.2^c^ (84)	< 0.01**
rs316246997		GG	GT	TT	
	P1	12.77 ± 2.1^a^ (118)	12.91 ± 2.14^a^ (319)	13.45 ± 1.75^b^ (264)	< 0.01**
	P2	12.81 ± 1.94^a^ (54)	13.07 ± 2.25^a^ (179)	14.36 ± 1.56^b^ (175)	< 0.01**
rs15913691		AA	AC	CC	
	P1	14.68 ± 1.5^a^ (57)	13.21 ± 1.93b (236)	12.82 ± 2.02c (427)	< 0.01**
	P2	14.82 ± 1.38^a^ (55)	13.73 ± 1.92c (119)	13.23 ± 2.13b (234)	< 0.01**
rs316044128		CC	CT	TT	
	P1	14.71 ± 1.61^a^ (49)	13.06 ± 2.12^b^ (236)	12.92 ± 1.91^b^ (416)	< 0.01**
	P2	14.85 ± 1.42^a^ (51)	13.4 ± 2.32^b^ (131)	13.41 ± 1.9^b^ (226)	< 0.01**

DF: the number of days following insemination until the last fertile egg was produced. Among genotypes within each SNP for each population, mean values bearing different letters indicate significant differences at **P < 0.01.

### *CYP2D6, WBP2NL, ESR1* and *TGFBR3* were up-regulated in hens with long duration-of-fertility trait

**Figure 4:**
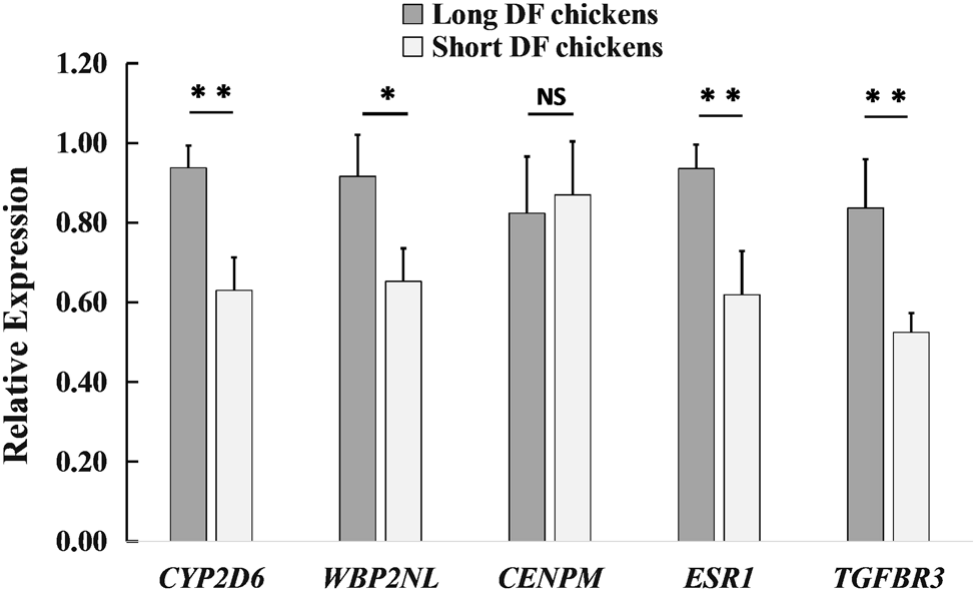
The *CYP2D6, WBP2NL, CENPM, ESR1* and *TGFBR3* mRNA relative expression levels in UVJ of the long duration-of-fertility trait (L-DF) hens and the short duration-of-fertility trait (S-DF) hens. * and ** indicate *P*-value respectively significance at the threshold levels of 0.05 and 0.01.

The above result shows the 27 GWAS SNPs were mapped to 7 genes according to their genomic position. Furtherly, 5 of these 7 genes were tested using qPCR. As presented in Fig. 4, the L-DF hens have significantly higher mRNA expression levels of *CYP2D6, WBP2NL, ESR1* and *TGFBR3* of UVJ tissues than the S-DF hens (P < 0.05).

## Discussion

In the present study, we performed GWAS to identify the critical SNPs or genes that affect the duration-of-fertility trait DF. The highlights of our study are as follows. First, the DF showed high individual variability even in populations of commercial strain and potentiality for genetic improvement. Second, 27 SNPs respectively located in three genomic linkage regions could serve as QTL (quantitative trait locus) or molecular markers for DF, while 4 QTL genes could be the key regulatory genes for further mechanism study of DF trait.

The capability of Hens sustainably laying fertile eggs for days or weeks post-mating in their breeding season^21^ was termed the duration-of-fertility^8,22^, it is an important economic trait in chick production when it has strong effects on fertile egg output and production costs including feeding costs, AI labor costs and so on^2,23^. In our studies, DF varies remarkably among individuals which ranged from 8.67 to 16.42 days (2.5–97.5%) with phenotypic variation coefficients 15% in the Jinghong commercial population. In the Jingfen commercial population, the DF ranged from 8.39 to 17.00 days (2.5–97.5%) with phenotypic variation coefficients 15%. These results presented here are similar to those of studies performed on other chicken strains, in which the DF was a medium repeatability trait and showed high individual variability among hens. In practice, DF was not only remarkably varied between individuals but also between breeds and strains. It means that DF-relative genetic variability was expected to exist. In hatching egg productionIn practice, DF is a determinant limitation by which the frequency of artificial insemination (AI) must be managed^5,16, 24^. For example, the DF of turkeys is quite a long period, lasting over one month. Therefore, artificial insemination only needs to be carried out once a month in the production process of fertile turkey eggs^22,25^. In some commercial lines, including chickens (White leghorn and Jinghong hens) and ducks (Anas platyrhyncha domestica), their DF characteristics are generally less than two weeks^5,12^. The pheno value of DF is 13.09 ± 2.01 days and 13.59 ± 2.05 days for the JHP and JFP populations respectively, in order to ensure a fertilization rate of over 90% for breeding eggs in production, artificial insemination always requires a high frequency of once a week. This indicates that the productivity of some of the hens had not yet been realized by the end of the observation period, when approximately 35% of the hens in our study were still laying fertile eggs 14 days after insemination. Above all, the DF trait has a large potential for genetic improvement and actual utilization value in hatching egg production.

The genome-wide scan is an effective approach that can be used to gain an understanding of the important markers and functional genes that affect complex quantitative traits. Therefore, we next performed a GWAS to uncover the critical SNPs or genes that affect the DF in hens using egg production data from Jing Hong chickens. Collectively, 27 SNPs were selected to be reached genome-wide significantly associated with DF in the commercial population of Chinese Jinghong hens. Out of them, the genotype effects on DF of 6 tag SNPs were further confirmed in the JHP and JFP with PCR-RFLP genotyping data. Based on the results of association analysis, it has been shown that the C allele at SNP rs312949642, A allele at SNP rs13864590, C allele at SNP rs312853002, T allele at SNP rs316246997, A allele at SNP rs15913691, and C allele at SNP rs316044128 in chromosome 1, 3 and 8 are the most potential candidate molecular genetic markers that can be used to improve the DF in marker-assisted selection programs.

Linkage disequilibrium plays a vital role in mapping genes that affect complex diseases and identifying associations among genetic markers and functional genes^26^. Understanding LD among SNP also avoids redundant inferences involving nonindependent genetic markers. The LD analysis revealed that the 27 significantly associated SNPs were in more or less significant LD with each other in three genome regions of chromosome 1, 3 and 8, which implies that these polymorphisms are associated with our studies on the DF trait. Three chromosomal regions (GGA1: 41Kb, GGA3: 39Kb and GGA8: 39Kb) were suggestively associated with DF. These candidate QTLs were first reported and six genes around these SNPs were annotated based on the ICGSC annotation of the Gallus gallus genome version 4.0, including the *CYP2D6*, *WBP2NL*, *CENPM*, *ESR1* and *TGFBR3* genes in chickens. Bakst et al. reported that the biological basis of duration-of-fertility is the capacity of hens to store a population of sperms in the oviduct for days or weeks throughout the period of egg production^11,27^. In order to store sperm, females possess specialized simple tubular invaginations, referred to as sperm storage tubules (SST), located in the uterovaginal junction (UVJ) mucosal folds where the spermatozoa are released for upward transport towards the infundibulum for ova fertilization^28^. They also indicated that the favorable mechanisms responsible for sperm storage were also functional in regulating the duration-of-fertility trait of hens. Intriguingly, our qPCR results showed that the chickens with long DF trait have significantly higher *CYP2D6*, *WBP2NL*, *ESR1* and *TGFBR3* expression levels in the UVJ tissue than the chickens with short DF trait. As there is no in-depth functional research about the above genes in chickens, we speculate that they may regulate DF via interacting with the reproductive system based on studies in humans. The *CYP2D6* gene encodes a member of the cytochrome P450 superfamily of enzymes, which are monooxygenases that catalyze many reactions in drug metabolism and synthesize cholesterol, steroids and other lipids^29^. Another gene *WBP2NL* is a sperm-specific WW domain-binding protein that promotes meiotic resumption and pronuclear development during oocyte fertilization^30^. The *ESR1* gene encodes an estrogen receptor and ligand-activated transcription factor^31^. Previous studies reported that the protein encoded by this gene regulates the transcription of many estrogen-inducible genes that play distinct roles in controlling growth, metabolism, sexual development, gestation, and other reproductive functions^32,33^. *TGFBR3* gene encodes the transforming growth factor (TGF)-beta type III receptor on the cell surface and endoplasmic reticulum. Enables several functions to participate in many important physiological processes to influence sexual maturity, such as gonadotropin-releasing hormone, vasopressin and oxytocin secretion^34^. In addition, acts upstream of or within several processes, including positive regulation of NF-kappaB transcription factor activity, positive regulation of transmembrane receptor protein serine/threonine kinase signaling pathway, and vasculogenesis. In summary, we speculate that variations and expression of these genes might indirectly or directly contribute to the susceptibility of chicken DF, and further validation is required in multiple experiments.

In conclusion, our present study demonstrates that the DF trait has a large potential for genetic improvement and actual utilization value in fertile egg production. Therefore, the GWAS performed in this study strongly suggested that the SNPs in chromosome 1, 3 and 8 could be considered as the genetic markers used to improve DF of hens. Moreover, four additional genes (*CYP2D6*, *WBP2NL*, *ESR1* and *TGFBR3*) identified by annotating 27 genome-wide significant SNPs could be considered as candidates associated with DF. Findings in our research provide new insight into the genetic basis of the duration of fertility in hens, while further functional validation is still needed in other chicken breeds or any other animal species.

## Materials and methods

### Ethics statement

All the hens involved in the study were housed and handled according to the recommendations in the Guide for the Care and Use of Laboratory Animals of the Ministry of Science and Technology of China and protocols approved by the Scientific Ethics Committee of Guilin Medical University (permit number: GLMC-IACUC-20241017). And the methods employed in this research were strictly adhered to ARRIVE guidelines (https://arriveguidelines.org). All efforts were made to minimize animal suffering.

### Animal management and data collection

A total of 701 healthy Chinese Jinghong breeding hens (Jinghong population, JHP) and 408 Chinese Jingfen breeding hens (Jingfen population, JFP) at 30 weeks old were obtained from the poultry farm of the Yukou Poultry Industry Co. Ltd (Beijing, P. R. China). These laying hens were used to routinely produce fertile eggs before the experiment. All birds were raised in individual cages and kept in identical light/dark cycles. They all had ad libitum access to clean water and a commercial diet until the end of the experiment.

Duration-of-fertility trait (DF, the number of days following insemination until the last fertile egg was produced) was measured in 3 continuous age periods (32–34 weeks, 35–37 weeks, and 38–40 weeks). Briefly, at the beginning of each period, all hens were artificially inseminated with 2.00 × 10^8^ sperms from pooled ejaculates collected from roosters. To prevent any undesirable effects of the interval between inseminations and oviposition on subsequent fertility, insemination was always performed in the afternoon. Eggs were collected and marked daily from the 2nd to 21st day after AI. Fertility was checked by candling eggs on the 10th day of incubation (dead embryos were considered fertile). Quantitative expressions of fertility phenotypic trait DF for every hen were presented as their mean value measured in the 3 periods. The hens with egg laying rate > 90% and the fertilization rate > 90% during the DF remained.

### Exploring SNPs associated with duration-of-fertility trait using GWAS

Upon completion of the above experiments, a total of 192 hens (the egg laying rate > 95% and the fertilization rate > 90% during the DF) were randomly separated from the Jinghong population (JHP) and assigned to a GWAS. 0.5 mL blood samples were collected from the wing veins of all hens and mixed with an anticoagulant containing 1.5% EDTA. DNA was extracted using the phenol chloroform method. The concentration of DNA samples was quantified using an ND-2000 spectrophotometer (NanoDrop, USA) and adjusted to be approximately 50 ng/μL. Then, each sample was genotyped using the 600K Affymetrix Axiom HD chicken genotyping array. A set of 552,395 scorable SNPs were revealed. Chromosomal positions for each SNP marker were obtained by referring to the chicken reference genome (ftp://ftp.ensembl.org/pub/release-73/fasta/gallus_gallus/dna/). These SNPs were mapped to positions on Gallus gallus chromosome GGA1 through GGA28 and -chromosome Z. Quality control (QC) and association analysis were performed using the TASSEL software package (version 5.0), in which SNPs selection required more than 5% minor allele frequency (MAF), and a 95% or greater genotype call rate. After that, 341,176 SNPs were finally selected for GWAS to investigate whether the effects of these quantitative trait loci are associated with DF. The number of filtered SNP markers per chromosome with known positions ranged from 220 on chromosome 16 to 62,807 on chromosome 1, and the average density of adjacent SNP markers is presented in Supplementary Table S1.

Then, a kinship matrix of the SNP markers data was generated by the kinship analysis procedure and served as a random effect in a mixed linear model. The model equation can be expressed as follows: y = µ + G + K + e, in which y is the vector of phenotypes, μ is the mean of the phenotypes, G is the genotypes, K is the kinship matrix, and e is the residual vector. At last, the threshold *P*-value of 5% genome-wide significant association with the duration-of-fertility measured trait DF was calculated based on the "linkage disequilibrium–adjusted" Bonferroni method. Linkage disequilibrium (LD) was evaluated using Tassel package. The SNPs were defined as a block when the first and last markers are in strong LD (D′ value > 0.6) with all intermediate markers. Based on the sum of the blocks and SNPs not in a block (inter-block SNPs), the hens were estimated to have 88,994 “independent” tests, so that the threshold P-values of 5% Bonferroni linkage disequilibrium–adjusted genome-wide significance was 5.62e−07 (0.05/88,994). The threshold of *P*-value for the significance of “suggestive association” that allows one time false positive effect in GWAS test was calculated based on the same method as noted above and it was 1.12e−05 (1/88,994)^21,35^.

For assessing the proportion of phenotypic variance explained (PVE) by the combined effect of the 27 suggested SNPs. A multiple regression model was constructed. The phenotypic and genotype data of SNPs were used as dependent and independent variables, respectively. The form of the model is Y = β_0_ + β_1_*SNP1 + β_2_*SNP2 + ⋯ + β_27_*SNP27 + δ, where Y is phenotypic data, SNP1, SNP2, …, SNP27 is the genotype data of 27 SNP, β_0_, β_1_, …, β_27_ is the corresponding regression coefficient and δ is the error term. The statistical software (R function “lm”) was used to fit the multiple regression model, and the goodness of fit index (R square value) of the model was obtained. The R square value means the proportion of phenotypic variance, which could be explained by the combined effect of the SNPs.

### Test the genotype effect of GWAS SNPs on duration-of-fertility using PCR-RFLP

To further test the association significance of the duration-of-fertility trait with the genotypes of SNPs suggested in GWAS, 6 SNPs (rs312949642, rs13864590, rs312853002, rs316246997, rs15913691, rs316044128) were genotyped using PCR-RFLP and association analysis for DF in the 701 hens of JHP and the 408 hens of JFP respectively. Briefly, DNA samples were amplified using specific PCR primers, and the PCR products were digested by restriction enzymes (Xmn I, Stu I, Ban I, Mse I, Mse I and Xcm I respectively) following the manufacturer^'^s instructions. The enzyme-digested PCR products were then separated by agarose gel electrophoresis and visualized by UV light. Genotypes of each sample were revealed in accordance with the restriction patterns. Association analyses were performed using 1-way ANOVA, and LSD (Least-Significant Difference) to compare mean values among different genes by pairs in SPSS statistics software (version 8.0; SAS Inst. Inc., Cary, NC). Quantitative expression of the data is presented as means ± SD, and values were considered significantly different with *P*-values less than 0.01.

### RNA isolation, cDNA synthesis and Quantitative Real-time PCR

In order to investigate the effects of the GWAS gene CYP2D6, WBP2NL, CENPM, ESR1 and TGFBR3 expression levels in uterovaginal junction (UVJ) on duration-of-fertility, the long DF (L-DF, n = 5) Jinghong hens and the short DF (S-DF, n = 5) Jinghong hens were euthanized by decapitation under anesthesia on the 13th day after AI. The UVJ mucosa was dissected immediately, adhering connective tissues were removed, and total RNA was extracted using Trizol reagent (Invitrogen, Foster City, CA, USA) according to the manufacturer’s protocol. One microgram of pooled RNA was used to synthesize cDNA by the EasyScript™ one-step gDNA Removal and cDNA Synthesis SurperMix (TansGen Biotech, Beijing). The mRNA levels were analyzed with quantitative real-time PCR (qRT-PCR) and were performed on a CFX96 Real-Time Detection System (Bio-Rad) using Quantifast™ SYBR Green PCP Kit (QIAGEN, Germany) with GAPDH used to normalize the relative abundance of mRNA in each reaction. Each sample was assayed in triplicate, and The analysis of mRNA expression level was calculated using 2^−ΔΔCt^ method.

### Supplementary Information

Supplementary Information.

## Data Availability

All data generated in this study are included in this published article and its Supplementary Information files.
