# Supplementary material for: Investigating the genetic determination of duration-of-fertility trait in breeding hens
Source: Sci Rep. 2024 Jun 27;14:14819. doi: 10.1038/s41598-024-65675-0 (PMC11211418; doi:10.1038/s41598-024-65675-0)
Supplement: Supplementary file 1 — Supplementary Information. [file 41598_2024_65675_MOESM1_ESM.pdf]

Investigating the genetic determination of duration-of-fertility trait in breeding hens

Wei Luo[1], Xishi Huang[1], Jingxuan Li[1], Lantao Gu[1*]

[1] Institute of Biotechnology of Guilin Medical University, Guilin, Guangxi, China.

[*]Corresponding author: 308497542@qq.com

Supplementary Materials

Table S1. Distributions for SNPs by chromosomes after quality control (QC);

Figure S1 Quantile-Quantile plot describing the deviation between observed and expected $-\log_{10}$ *P*-values.

Table S1. Distributions for SNPs by chromosomes after quality control (QC)

Chromosomes	SNP No. remained after QC	Average Density (SNPs/Mbp)
GGA 1	62807	321.70
GGA 2	40690	273.50
GGA 3	35957	325.56
GGA 4	26971	299.12
GGA 5	19695	330.78
GGA 6	12514	358.26
GGA 7	14930	412.48
GGA 8	11516	400.92
GGA 9	10122	432.24
GGA 10	10275	517.58
GGA 11	8898	459.11
GGA 12	9558	481.66
GGA 13	7664	431.65
GGA 14	6968	460.13
GGA 15	6384	504.80
GGA 16	220	444.48
GGA 17	5592	546.87
GGA 18	5704	511.11
GGA 19	4902	491.14
GGA 20	5664	396.84
GGA 21	5330	790.85
GGA 22	2158	529.99
GGA 23	3788	664.09
GGA 24	4900	776.52
GGA 25	1514	691.80
GGA 26	3645	699.10
GGA 27	3300	637.53
GGA 28	3301	697.89
Z	6118	76.31
LGE1	31	48.59
LGE2	60	70.24

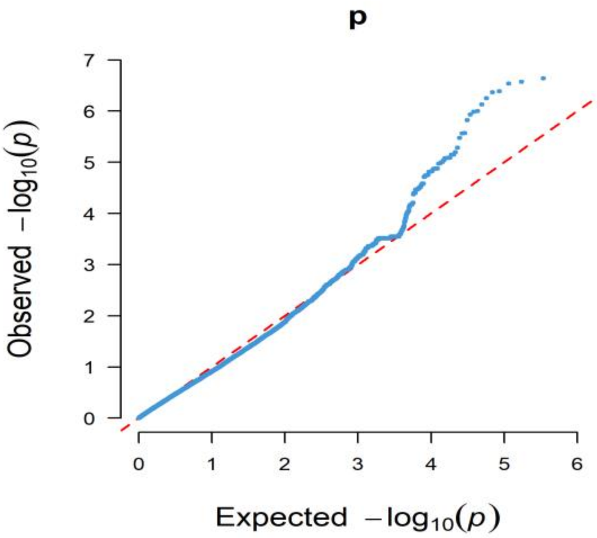

Figure S1 Quantile-Quantile plot describing the deviation between observed and expected –$\log_{10}$ *P*-values. The red solid diagonal line indicates the expected values and the blue plots stand for the observed values from genome wide association study.
